# Development and optimization of a female-specific Biomechanical model for biodynamic response analysis: a comparison with male biomechanical models

**DOI:** 10.1038/s41598-026-36165-2

**Published:** 2026-01-22

**Authors:** Veeresalingam Guruguntla, Bonda Atchuta Ganesh Yuvaraju, Thota S. S. Bhaskara Rao, G. S. Pradeep Ghantasala, Pellakuri Vidyullatha, Hari Prasadarao Pydi

**Affiliations:** 1 Department of Mechanical Engineering, Madanapalle Institute of Technology & Science (MITS), Deemed to be University, Madanapalle, Andhra Pradesh India; 2 School of Mechanical Engineering, Vellore Institute of Technology Chennai, Chennai, Tamil Nadu 600127 India; 3 Department of Computer Science and Engineering, Alliance School of Advanced Computing, Alliance University, Bengaluru, India; 4 Department of Computer Science and Engineering, Koneru Lakshmaiah Education Foundation, Vaddeswaram, Guntur, Andhra Pradesh India; 5 Department of Mechanical Engineering, Bule Hora University, Bulehora, Ethiopia

**Keywords:** Whole body vibration, Human body modelling, Experimental verification, Biodynamic responses, Firefly algorithm, Optimization, Sensitivity analysis, Anatomy, Computational biology and bioinformatics, Engineering, Physiology

## Abstract

Whole-body vibration exposure is a critical factor affecting human health and comfort, particularly for individuals operating on/off-road vehicles. Prior studies have focused on male biomechanical models. This study intentions to develop a new female-specific biomechanical model to analyze and optimize biodynamic responses under vertical vibration conditions. The objective is to introduce a ten degrees-of-freedom (dofs) biomechanical model tailored for the female body, considering the average weight of human beings. The new model has compared against existing male-oriented models to evaluate its effectiveness. The female body is divided into ten key segments: head, pelvis thorax, abdomen, left upper arm, left hand, left forearm, right upper arm, right forearm, and right hand. Mechanical properties are adjusted based on female-specific mass distribution, stiffness, and damping characteristics. The Firefly Algorithm is used for parameter optimization. The biodynamic responses, including seat-to-head transmissibility, apparent mass, and driving point mechanical impedance, are evaluated and compared with previous male models. The optimized female model exhibits distinct biodynamic response characteristics due to anatomical and biomechanical differences. The goodness of fit analysis indicates improved predictive accuracy for female subjects, suggesting the necessity for gender-specific modelling in vibration analysis.

## Introduction

The human body is a complicated system with distinct biomechanical properties that alternate relying at the environment and the individual. Activities consisting of drilling, mining, construction, and using a vehicle can reveal humans to vibrations, which may be uncomfortable and harmful to their health^1^. Whole-frame vibration (WBV) has been linked in research to musculoskeletal conditions, exhaustion, and reduced performance^2–9^.

Experiments on living humans and cadavers have traditionally been used to observe human biomechanical properties^10^. This research quantifies WBV reactions and compares them with biodynamic models. Studies have found that a 4-degrees-of-freedom (dofs) model provides a reasonable approximation of experimental transmissibility ratios^11^. However, standing posture, knee angles, and other body conditions also influence results^12^.

Since direct experimentation on humans is expensive and potentially unsafe, simulation techniques have become the preferred approach for evaluating biomechanical responses. Lumped parameter modelling (LPM) allows researchers to analyze how vibrations commencing discrete sources-such as vehicles and power tools-affect human dynamics^13^.

Over time, various LPM-based models have been proposed. A simple 2-dofs model, where the head and torso are connected by springs and dampers, was first introduced for shipboard applications^14^ and later adapted for vehicle ride analysis^15–19^. More advanced models, such as the 4-dofs^16^ and 7-dofs models^17–21^, have further refined human body segmentation to improve accuracy^22,23^. A 5-dofs model was specifically designed for seat comfort but lacked the ability to assess seat cushion materials. Some models incorporated backrests, lateral vibrations, and fore-aft motion to provide a more detailed recognizing of human response to vibrations^17–24^.

A 9-dofs model^25,26^ has been used to study the impact of high-speed train vibrations on internal organs, while an 11-dofs model examined the effects of lateral and longitudinal excitations on pregnant women^27^. These studies highlight the importance of accurately modelling human body dynamics.

Most existing models simplify the human structure by combining multiple segments into one, reducing accuracy in biomechanical response predictions. Although some models attempt to increase segmentation, they still do not fully represent the complexities of human anatomy. This study aims to develop a female biomechanical model that closely mimics the real human body structure.

The proposed model applies Newton’s second law to derive dynamic equations of motion, applying the Firefly Algorithm (FA) for optimization. The optimized parameters will help improve ergonomic seating, vehicle safety, and test dummy design for crash analysis. Additionally, LPM techniques will be used to study energy transmission through muscles and segment deformation under vertical vibrations.

This research is conducted in two phases. First, a novel 10-dofs female biomechanical model is developed and optimized using FA. Second, previous models-including the Allen 2-dofs, Wan and Schimmels 4-dofs, Bai et al. 4-dofs, Darling et al. 7-dofs, and Boileau et al. experimental models are optimized for comparison. Modal and sensitivity analyzes are performed to assess the effects of adapting parameters on biodynamic responses and compared with the male biomechanical model.

Research published in^28–35^ investigated the effect of gender on biodynamic responses during exposure to vertical vibration using whole-body vibration training machines. The study, which involved 40 subjects (20 females and 20 males), examined vertical vibrations ranging from 20 to 45 Hz. Findings indicated that males exhibited higher apparent mass compared to females, while females demonstrated higher transmissibility toward the head in altogether directions. These differences were attributed to physiological and anatomical variations, such as body mass distribution, muscle composition, and structural stiffness.

Females generally exhibit slightly lower resonance frequencies due to lower body mass and different tissue composition, which affects parameters like Seat-to-Head Transmissibility (STHT) by shifting the peak response to a lower frequency. Furthermore, because of their decreased body weight and specific mechanical impedance response, females commonly exhibit smaller amplitudes in Apparent Mass (AM) and Driving Point Mechanical Impedance (DPMI). According to positive research, females may have more damping in soft tissues, which can decrease their peak transmissibility. Additionally, how the vibrations travel via the frame is affected by modifications in posture and spinal curvature, and research advise that women can be greater liable to lower-frequency vibrations, which can have an effect on their consolation and degree of weariness.

These discrepancies had been ultimately investigated in^28–35^, which discovered that despite the fact that women absorbed more basic vibration power than males with comparable frame measurements, their top vibration power absorption (VPA) turned to decrease. This means that due to variations in the distribution and make-up of body fat, males take in vibrations with a better peak depth even as females take in more energy overall. These consequences highlight how important it is to take gender into consideration while growing equipment and workspaces as a way to effectively lessen the fitness issues related to vibration.

Seat and vehicle layout are extensively impacted through the damping disparities among male and female biodynamic reactions, in particular with regards to enhancing comfort, safety, and vibration avoidance. Seat cushions and suspensions must be made with adaptive damping substances to deal with each decrease and better damping responses due to the fact females commonly have better damping in soft tissues. Additionally, seat stiffness has to be capable of being adjusted, probably the use of smart substances or active suspension structures that reply to the features of the rider, as females take in more vibration strength but display decreased peak VPA. Suspension and seat dampening structures have to be adjusted to lessen vibrations, specifically for the duration of extended rides, seeing that females are more sensitive to lower-frequency vibrations (under 10 Hz). In order to reduce discomfort added on through extended vibration exposure, gender-inclusive seat designs have to additionally consist of optimum lumbar support, contouring, and seat inclination. Automated seat changes or adjustable damping settings should enhance long-time period comfort due to the fact studies indicates that women may also sense more worn-out and uncomfortable. Additionally, biodynamic variations affect crashworthiness and affect absorption, which requires similar observation of gender-based biomechanically-based adaptive protection measures like airbag deployment and seatbelt tensioning. All matters considered, those effects emphasize how critical it is to create vehicles and places of work that consider the biodynamic reactions of each male and females so that you can achieve growth consolation and protection.

Although parameter adjustments to existing male-oriented models may seem sufficient, empirical studies indicate that simple scaling or stiffness-mass adjustments cannot reproduce the unique resonance frequencies and damping characteristics observed in females. The biomechanical responses of the female body are not merely scaled-down versions of the male system; they arise from structural and physiological differences, such as higher soft tissue damping, lower bone stiffness, a unique pelvic geometry, and different mass distribution ratios between the upper and lower body segments. These nonlinear coupled effects alter modal behaviour and energy transfer pathways, thus requiring a separate biomechanical framework. Developing female-specific models can more accurately characterize these gender-driven dynamic features, thereby improving the predictive accuracy of comfort analysis, ergonomic design, and safety assessments.

This study considers only vertical (z-axis) vibrations while intentionally neglecting lateral (x-axis) and longitudinal (y-axis) components. This simplification is intended to focus on the primary vibration direction experienced by seated occupants, which is generally the main cause of discomfort and health risks in off-road and vehicular environments. Although this assumption improves the model’s operability and allows for fine optimization of vertical biodynamic parameters, it limits the model’s generalizability under multi-axis vibration conditions, such as those encountered in aircraft, heavy machinery, or uneven terrain. Future extensions of the model may incorporate coupled lateral and longitudinal motions to capture cross-axis interactions and enhance the prediction capability for complex vibration environments.

The overall workflow of this study includes four consecutive stages: model development, parameter estimation, optimization, and validation. First, we developed a lumped-parameter biomechanical model with 10 degrees of freedom (DOF) to simulate the motion of the female body under vertical vibration. The model represents the head, chest, abdomen, pelvis, and upper limbs as discrete mass blocks, connected by spring-damper elements to capture the stiffness and damping characteristics between segments. The initial values for mass, stiffness, and damping were derived from anthropometric literature and validated male models, and were then adjusted using female-specific scaling factors based on female segment mass distribution and soft tissue characteristics. These parameters serve as the starting point for the Firefly Algorithm (FA) optimization, which aims to minimize the sum of squared errors of the model-predicted biomechanical responses (i.e., seat-to-head transmissibility (STHT), apparent mass (AM), and driving-point mechanical impedance (DPMI)) within the 0.5–20 Hz frequency range. Subsequently, the optimized parameters were validated against experimental data from seated female subjects exposed to controlled vertical sinusoidal excitations to ensure their physiological consistency and accuracy. Finally, using goodness-of-fit (GOF) metrics, modal analysis, and sensitivity evaluation, the performance of the optimized model was compared with several established male biomechanical models, demonstrating that the proposed female-specific model possesses superior predictive capability and reliability.

## Modelling

The human body has a dynamic, complicated entity this is too complicated to be immediately examined. A mathematical model of female biomechanics is created to resource in each quantitative and qualitative examination. With an emphasis on dynamic equations of motion (EOMs) below vertical vibration circumstances, this phase affords a 10-degrees-of-freedom (dofs) biomechanical model for a female sitting occupant. Although numerous biomechanical models have been developed to study whole-body vibration, most existing frameworks have some inherent limitations. Many models simplify the human structure by aggregating multiple anatomical structures (e.g., chest, abdomen, and pelvis) into a single lumped segment, thereby neglecting the coupling between segments and local resonance effects. Other models heavily rely on male anthropometric data, assuming that female biomechanics can be represented through linear mass scaling or stiffness adjustments, which fails to capture nonlinear soft tissue damping and gender-specific resonance behaviours. Additionally, some early models employed low degrees of freedom (2–7 DOF), limiting their ability to simulate realistic posture-related responses or assess energy transfer between different body segments. Some studies also lack optimization-based parameter calibration, resulting in limited agreement with experimental results.

This study effectively addresses the aforementioned shortcomings by constructing a 10-degree-of-freedom biomechanical model specifically for females. The model can clearly represent the main segments of the upper body and limbs. The mass, stiffness, and damping parameters of each segment are derived from female-specific anthropometric data and experimentally validated vibration responses, thereby ensuring the physiological realism of the model. In addition, this study uses the Firefly Algorithm (FA) for parameter optimization, further improving the model’s accuracy and minimizing the deviation between the model predictions and experimental data in the frequency range of 0.5–20 Hz. By integrating high-resolution segmentation, gender-specific parameterization, and advanced metaheuristic optimization algorithms, this study constructs a comprehensive and validated model that bridges the gap between simplified male models and the complex biomechanics of females.

Assumptions for Female Biomechanical Model:

- The human body is represented as a lumped-mass spring-damper system, in which rigid segments are interconnected through elastic and damping elements.
- This model only considers vertical (z-direction) vibrations and neglects fore-aft and lateral movements.
- Each body segment (head, torso, pelvis, etc.) is assumed to be a rigid body with no internal deformation.
- Female subjects maintain a seated posture and keep a constant posture throughout the analysis.
- The interaction between the seat and the body is continuous and represented by equivalent stiffness and damping parameters.
- Depending on the experimental setup, the subject’s feet can either be supported on the floor or ignored.
- The model assumes that all spring-damper elements behave linearly within the range of vibration amplitudes.
- Assume that the backrest and seat cushion have constant mechanical properties during vibration exposure.
- Assume that the total weight of an adult female (weight at the 50th percentile) is approximately 60–70 kg.
- Vibration input is applied to the bottom of the seat in the form of harmonic or random vertical excitations.
- The frequency range of interest is typically between 0.5 Hz and 20 Hz, which is relevant to whole-body vibration studies.
- Ignore inter-subject differences (age, body type, health status) and assume the subjects are of average build.
- The model is considered symmetric about the sagittal plane, thus lateral asymmetry is neglected.

### Equation of motion (EOMs)

**Fig. 1:**
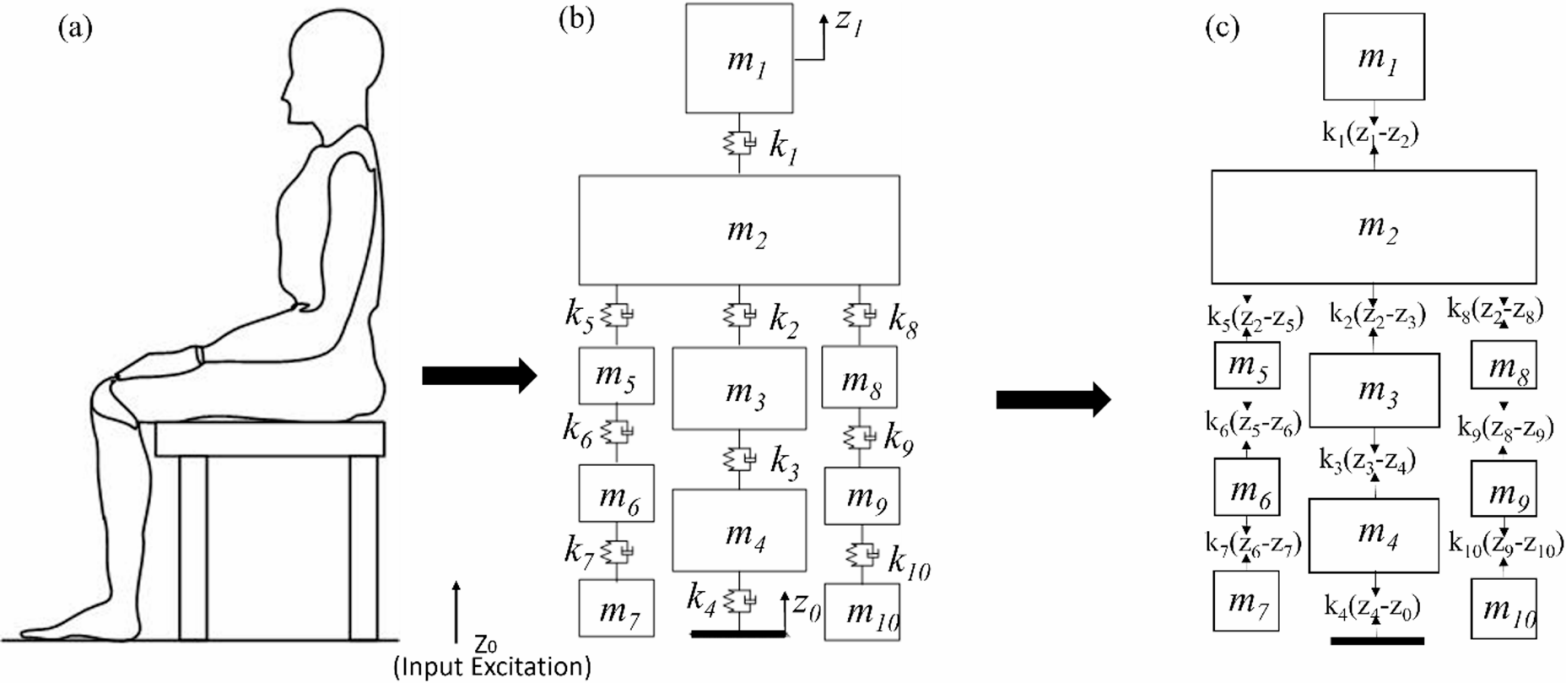
(**a**) Experimental posture (**b**) Proposed a 10-dofs biomechanical model (**c**) Free body diagram for a proposed model.

The overall biomechanical modelling framework used in this work are illustrated in Fig. 1. The schematic picture of female human-being while experimenting for data collection is presented in Fig. 1(a), forming the basis of analysis. From this posture, a 10-dofs biomechanical model for a seated occupant was developed to represent the lower-limb and trunk segments, as shown in Fig. 1(b). The human body is conceptualized into segments including the abdomen, head, thorax, pelvis, forearms, upper arms, and hands, while the legs are excluded from this analysis. These segments are connected through springs and dampers to replicate real human biomechanics.

In Fig. 1 (b & c), *m*_i_ indicates mass of corresponding segment, *k*_i_ and *c*_i_ are stiffness and damping of in-between adjacent segments (*i* = 1 to 10). The input displacement from the seat to the body is represented as *z*_o_, whereas *z*_i_ (*i* = 1 to 10) signifies the vertical displacement of each body segment. By pertaining Newton’s second law to each segment, as depicted in Fig. 1(c) (To avoid the ambiguity only spring forces are presented, although damping forces are also shown), the equations of motion (EOMs) are derived as follows.

1 $${m_1}{\ddot {z}_1}+{k_1}({z_1} - {z_2})+{c_1}({\dot {z}_1} - {\dot {z}_2})=0$$

2 $$\begin{gathered} {m_2}{{\ddot {z}}_2}+{k_1}({z_2} - {z_1})+{c_1}({{\dot {z}}_2} - {{\dot {z}}_1})+{k_2}({z_2} - {z_3})+{c_2}({{\dot {z}}_2} - {{\dot {z}}_3}) \hfill \\ +{k_5}({z_2} - {z_5})+{c_5}({{\dot {z}}_2} - {{\dot {z}}_5})+{k_8}({z_2} - {z_8})+{c_8}({{\dot {z}}_2} - {{\dot {z}}_8})=0 \hfill \\ \end{gathered}$$

3 $${m_3}{\ddot {z}_3}+{k_2}({z_3} - {z_2})+{c_2}({\dot {z}_3} - {\dot {z}_2})+{k_3}({z_3} - {z_4})+{c_3}({\dot {z}_3} - {\dot {z}_4})=0$$

4 $${m_4}{\ddot {z}_4}+{k_3}({z_4} - {z_3})+{c_3}({\dot {z}_4} - {\dot {z}_3})+{k_4}({z_4} - {z_0})+{c_4}({\dot {z}_4} - {\dot {z}_0})=0$$

5 $${m_5}{\ddot {z}_5}+{k_5}({z_5} - {z_2})+{c_5}({\dot {z}_5} - {\dot {z}_2})+{k_6}({z_5} - {z_6})+{c_5}({\dot {z}_5} - {\dot {z}_2})=0$$

6 $${m_6}{\ddot {z}_6}+{k_6}({z_6} - {z_5})+{c_6}({\dot {z}_6} - {\dot {z}_5})+{k_7}({z_6} - {z_7})+{c_7}({\dot {z}_6} - {\dot {z}_7})=0$$

7 $${m_7}{\ddot {z}_7}+{k_7}({z_6} - {z_7})+{c_7}({\dot {z}_6} - {\dot {z}_7})=0$$

8 $${m_8}{\ddot {z}_8}+{k_8}({z_8} - {z_2})+{c_8}({\dot {z}_8} - {\dot {z}_2})+{k_9}({z_8} - {z_9})+{c_9}({\dot {z}_8} - {\dot {z}_9})=0$$

9 $${m_9}{\ddot {z}_9}+{k_9}({z_9} - {z_8})+{c_9}({\dot {z}_9} - {\dot {z}_8})+{k_{10}}({z_9} - {z_{10}})+{c_{10}}({\dot {z}_9} - {\dot {z}_{10}})=0$$

10 $${m_{10}}{\ddot {z}_{10}}+{k_{10}}({z_{10}} - {z_9})+{c_{10}}({\dot {z}_{10}} - {\dot {z}_9})=0$$

The combined EOMs for the entire system can be formulated as:

11 $${M_{10 \times 10}}{\ddot {z}_{10 \times 1}}+{C_{10 \times 10}}{\dot {z}_{10 \times 1}}+{K_{10 \times 10}}{z_{10 \times 1}}={f_{{z_{10 \times 1}}}}$$

Where, the Elements of stiffness and damping matrix and force vector are.

$$M=\left[ {\begin{array}{*{20}{c}} {{m_1}}&0&0&0&0&0&0&0&0&0 \\ 0&{{m_2}}&0&0&0&0&0&0&0&0 \\ 0&0&{{m_3}}&0&0&0&0&0&0&0 \\ 0&0&0&{{m_4}}&0&0&0&0&0&0 \\ 0&0&0&0&{{m_5}}&0&0&0&0&0 \\ 0&0&0&0&0&{{m_6}}&0&0&0&0 \\ 0&0&0&0&0&0&{{m_7}}&0&0&0 \\ 0&0&0&0&0&0&0&{{m_8}}&0&0 \\ 0&0&0&0&0&0&0&0&{{m_9}}&0 \\ 0&0&0&0&0&0&0&0&0&{{m_{10}}} \end{array}} \right]$$

$$K=\left[ {\begin{array}{*{20}{c}} {{k_1}}&{ - {k_1}}&0&0&0&0&0&0&0&0 \\ { - {k_1}}&{{k_1}+{k_2}+{k_5}+{k_8}}&{ - {k_2}}&0&{ - {k_5}}&0&0&{ - {k_8}}&0&0 \\ 0&{ - {k_2}}&{{k_2}+{k_3}}&{ - {k_3}}&0&0&0&0&0&0 \\ 0&0&{ - {k_3}}&{{k_3}+{k_4}}&{ - {k_4}}&0&0&0&0&0 \\ 0&0&0&{ - {k_4}}&{{k_5}+{k_6}}&{ - {k_6}}&0&0&0&0 \\ 0&0&0&0&{ - {k_6}}&{{k_6}+{k_7}}&{ - {k_7}}&0&0&0 \\ 0&0&0&0&0&{ - {k_7}}&{{k_7}}&0&0&0 \\ 0&0&0&0&0&0&0&{{k_8}+{k_9}}&{ - {k_9}}&0 \\ 0&0&0&0&0&0&0&{ - {k_9}}&{{k_9}+{k_{10}}}&{ - {k_{10}}} \\ 0&0&0&0&0&0&0&0&{ - {k_{10}}}&{{k_{10}}} \end{array}} \right]$$

$$C=\left[ {\begin{array}{*{20}{c}} {{c_1}}&{ - {c_1}}&0&0&0&0&0&0&0&0 \\ { - {c_1}}&{{c_1}+{c_2}+{c_5}+{c_8}}&{ - {c_2}}&0&{ - {c_5}}&0&0&{ - {c_8}}&0&0 \\ 0&{ - {c_2}}&{{c_2}+{c_3}}&{ - {c_3}}&0&0&0&0&0&0 \\ 0&0&{ - {c_3}}&{{c_3}+{c_4}}&{ - {c_4}}&0&0&0&0&0 \\ 0&0&0&{ - {c_4}}&{{c_5}+{c_6}}&{ - {c_6}}&0&0&0&0 \\ 0&0&0&0&{ - {c_6}}&{{c_6}+{c_7}}&{ - {c_7}}&0&0&0 \\ 0&0&0&0&0&{ - {c_7}}&{{c_7}}&0&0&0 \\ 0&0&0&0&0&0&0&{{c_8}+{c_9}}&{ - {c_9}}&0 \\ 0&0&0&0&0&0&0&{ - {c_9}}&{{c_9}+{c_{10}}}&{ - {c_{10}}} \\ 0&0&0&0&0&0&0&0&{ - {c_{10}}}&{{c_{10}}} \end{array}} \right]$$

$${\{ z\} _{10 \times 1}}={\left[ {\begin{array}{*{20}{c}} {{z_1}}&{{z_2}}&{{z_3}}&{{z_4}}&{{z_5}}&{{z_6}}&{{z_7}}&{{z_8}}&{{z_9}}&{{z_{10}}} \end{array}} \right]^T};$$

$${f_{y41{\text{ }}}}={k_{40}}z+{c_{40}}\dot {z}$$

wherever *M*, *K* and *C* are overall stiffness, mass, and damping matrices, correspondingly; *f*_*z*_ and *z* are the input force and displacement vector transferred from seat.

Upon accepting the solution of Eq. (11) as *z* = *Ze*^*jωt*^, Eq. (11) could be transformed into the frequency domain as

12 $$( - {\omega ^2}M+j\omega C+K)Z={F_Z}$$

where the response vector {*Z*} and force vector {*F*_*Z*_} are in the complex form as

13 $${Z_i}=Z_{i}^{{}}+jZ_{i}^{{}}$$

with subscript *i* = 1 to 10.

and,

14 $${F_Z}={\left[ {\begin{array}{*{20}{c}} 0&0&0&{({k_4}+j\omega {c_4}){Z_0}}&0&0&0&0&0&0 \end{array}} \right]^T}$$

Once segregating the real and imaginary terms, Eq. (14) might be written as.

$${F_Z}={\left[ {\begin{array}{*{20}{c}} 0&0&0&{{k_4}}&0&0&0&0&0&0 \\ 0&0&0&{{c_4}}&0&0&0&0&0&0 \end{array}} \right]^T}\left[ {\begin{array}{*{20}{c}} 1 \\ {j\omega } \end{array}} \right]{Z_0}$$

On combining Eq. (12), Eq. (13), and Eq. (14) overall response vector {*Z*} could be written as

15 $$Z={A^{-1}}B\left[ {\begin{array}{*{20}{c}} 1 \\ {j\omega } \end{array}} \right]{Z_0}$$

where $$A= - {\omega ^2}M+j\omega C+K;$$ and$$B={\left[ {\begin{array}{*{20}{c}} 0&0&0&{{k_4}}&0&0&0&0&0&0 \\ 0&0&0&{{c_4}}&0&0&0&0&0&0 \end{array}} \right]^T};$$.

Biodynamic responses provide crucial insights into human body behaviour, including critical frequencies of body components and modes of excitation^18^. In this section, Eq. (15) is usage has to formulate essential biodynamic responses: seat-to-head transmissibility (STHT), driving point mechanical impedance (DPMI), and apparent mass (AM).

The transfer function for STHT has the ratio of the displacement for a head to the displacement at seat-buttock interface expected to forced vibration. Through reference to Fig. 1(b) and from Eq. (15), it could be written as^18^

16 $$STHT=\frac{{{Z_1}}}{{{Z_0}}}$$

where *Z*_*0*_ and *Z*_*1*_ are input and head displacement, correspondingly.

The transfer function for DPMI explains as a ratio of force to velocity at driving location (i.e., pelvis) at identical frequency and from Fig. 1(b), it might stated as^18^

17 $$DPMI=\frac{{{F_4}}}{{{v_4}}}$$

where *F*_*4*_ and *v*_*4*_ are the force and velocity at a driving point (i.e., pelvis), correspondingly.

The transfer function for AM may denote as a ratio of force to acceleration at driving location (i.e., pelvis) at identical frequency and could express as^18^

18 $$AM=\frac{{{F_4}}}{{{a_4}}}$$

where *F*_*4*_ and *a*_*4*_ are force and acceleration at the driving point (i.e., pelvis), correspondingly.

## Objective function and constraints

The biomechanical system could be optimized using various parameters, but the most influential ones are the biodynamic characteristics: driving point mechanical impedance (DPMI), seat-to-head transmissibility (STHT), and apparent mass (AM). The objective function aims to minimize the sum of squared errors between biodynamic parameters obtained from real human experiments^28^ and those predicted by the proposed model. This can be expressed as:

9 $$\hbox{min} (F)=\mathop \Sigma \limits_{{i=1}}^{N} {\lambda _1}.{U_1}+{\lambda _2}.{U_2}+{\lambda _3}.{U_3}$$

with,

${U_1}={(STH{T_e}({f_i}) - STH{T_p}({f_i}))^2}$ at *i*^th^ frequency (*i* = 0.5–20 Hz).

${U_2}={(DPM{I_e}({f_i}) - DPM{I_p}({f_i}))^2}$ at *i*^th^ frequency (*i* = 0.5–20 Hz).

${U_3}={(A{M_e}({f_i}) - A{M_p}({f_i}))^2}$ at *i*^th^ frequency (*i* = 0.5–20 Hz).

where ‘*N*’ is a number of iterations, ‘λ’ is weighting function (Σλ_*j*_ = 1), subscripts ‘*e*’ and ‘*p*’ stand for an experimental and proposed model, correspondingly. Subsequently the human being is maximum sensitive at lower frequencies of vibration^19^. Therefore, the biodynamic parameters are expected in the frequency range (0.5–20) Hz.

The choice of a frequency range of 0.5–20 Hz is intended to match the main exposure spectrum of seated vehicle occupants’ vibrations and the bandwidth of experimental data used for model validation. Numerous studies have shown that the human body is most sensitive to whole-body vibrations below 20 Hz, with the most critical resonance modes of the pelvis, spine, and head-neck system typically occurring between 3 Hz and 6 Hz^18,19,28^. Frequencies above 20 Hz have little effect on perceived discomfort and are usually attenuated by the seat structure and soft tissues. Additionally, the experimental data sets used for parameter fitting and validation^28^ were also measured within this frequency range, ensuring comparability between simulated and observed biomechanical responses. Therefore, the 0.5–20 Hz range represents both the physiologically relevant working range for seated occupants and the empirically supported range of existing measurement data.

where:

- ’*i*’ represents the frequency index (ranging from 0.5 to 20 Hz),
- ‘N’ is the number of iterations,
- ‘λ’ is a weighting function such that subscripts ‘e’ and ‘p’ denote experimental and proposed model values, respectively.

Since humans are most sensitive to lower-frequency vibrations^19^, the biodynamic parameters are evaluated in the 0.5–20 Hz frequency range.

To improve the convergence rate and minimize the sum of squared errors, numerous stiffness, mechanical parameters-mass, and damping are treated as constraints. These constraints provide a rational basis for selecting model parameters and optimization techniques. The constraint equation is given as:

10 $$\left\{ \begin{gathered} \sum\limits_{{i=1}}^{{10}} {{m_i}} =54{\text{ kg}} \hfill \\ {{\mathrm{m}}_{\mathrm{5}}}{\mathrm{=}}{{\mathrm{m}}_{\mathrm{8}}}{\mathrm{,}}{{\mathrm{m}}_{\mathrm{6}}}{\mathrm{=}}{{\mathrm{m}}_{\mathrm{9}}}{\mathrm{,}}{{\mathrm{m}}_{\mathrm{7}}}{\mathrm{=}}{{\mathrm{m}}_{{\mathrm{10}}}} \hfill \\ {{\mathrm{k}}_{\mathrm{5}}}{\mathrm{=}}{{\mathrm{k}}_{\mathrm{8}}}{\mathrm{,}}{{\mathrm{k}}_{\mathrm{6}}}{\mathrm{=}}{{\mathrm{k}}_{\mathrm{9}}}{\mathrm{,}}{{\mathrm{k}}_{\mathrm{7}}}{\mathrm{=}}{{\mathrm{k}}_{{\mathrm{10}}}} \hfill \\ {{\mathrm{c}}_{\mathrm{5}}}{\mathrm{=}}{{\mathrm{c}}_{\mathrm{8}}}{\mathrm{,}}{{\mathrm{c}}_{\mathrm{6}}}{\mathrm{=}}{{\mathrm{c}}_{\mathrm{9}}}{\mathrm{,}}{{\mathrm{c}}_{\mathrm{7}}}{\mathrm{=}}{{\mathrm{c}}_{{\mathrm{10}}}} \hfill \\ 100{\text{ N}}{{\mathrm{m}}^{{\mathrm{-1}}}}<{k_{as}}<300000{\text{ N}}{{\mathrm{m}}^{{\mathrm{-1}}}} \hfill \\ 500{\text{ Ns}}{{\mathrm{m}}^{{\mathrm{-1}}}}<{c_{as}}<4000{\text{ Ns}}{{\mathrm{m}}^{{\mathrm{-1}}}} \hfill \\ \end{gathered} \right\}$$

where:

- ‘*m*’ represents the mass of each body segment,
- *k*_as_, *c*_as_ denote the stiffness and damping for adjacent segments, correspondingly.

Experimental data for an seated occupants^28^ determine the mass constraints, while the segmentation follows the approach in^29^. The stiffness and damping ranges are based on values from^19^.

The initial mass, stiffness, and damping parameters before optimization were determined by combining estimated values from the literature with proportionate scaling based on validated male biomechanical models. First, the mass of each segment was estimated according to average adult female anthropometric data, referencing the segment percentage distributions reported in references^19,29^. The initial values of stiffness and damping coefficients were derived from experimental studies on seated human biomechanical responses^18–21,25^ and then scaled according to the ratios of female to male segment mass and tissue composition (typically mass 0.85–0.90, damping 1.1–1.3, reflecting greater soft tissue attenuation in females). This hybrid approach ensures that all initial parameters remain within physiologically reasonable ranges, providing a stable starting point for the Firefly Algorithm optimization. Moreover, the literature-based initialization method minimizes the risk of converging to non-physical local minima and improves numerical stability during the iterative parameter adjustment process.

## Optimization technique

The Firefly Algorithm (FA) is a metaheuristic optimization method exploited by Xin-She Yang in 2008, inspired through the bioluminescent behaviour for fireflies. Fireflies use their flashing lights to attract mates or prey, and this natural phenomenon is simulated in FA to solve optimization problems. The algorithm is particularly effective in handling nonlinear, multimodal, and complex optimization problems across various disciplines^30–33^.

FA is based on three key rules: (1) all fireflies are unisex and can be attracted to each other, (2) the attractiveness of a firefly is directly proportional to its brightness, which is correlated to the objective function being optimized, and (3) the brightness reductions as the distance among fireflies increases. Less bright fireflies move concerning brighter ones, leading to convergence towards an optimal solution.

One of the principal benefits of FA is its high convergence rate, which enables it to quickly reach near-optimal solutions. Unlike traditional optimization methods, FA does not require gradient information and can effectively escape local optima due to its randomization feature. Because of this, it can be used to explain a diversity of scientific and engineering issues. Power systems optimization, image processing, wireless sensor networks, structural design, and smart manufacturing are just a few of the domains where FA has been successfully used. Additionally, it has been applied to biological fields like human motion analysis and prosthesis design optimization. FA has also been used for feature selection and hyperparameter adjustment in machine learning, scheduling, and finance^28–35^.

FA is widely used; however, it hasn’t been used to maximize the segmental qualities of female humans yet. FA may be essential in enhancing sports performance analysis, rehabilitation models, and ergonomic designs due to the physiological variations between male and female biomechanical structures^28–35^. Researchers can enhance the precision of biomechanical simulations and create more correct models for motion evaluation specific to girls through utilising its resilience and versatility.

With its competence to strike a stability among exploration and exploitation, the Firefly Algorithm remains a robust tool in current optimization and indicates promise in resolving difficult real-world issues. FA is a novel method for optimizing the biomechanical model of females, but, because it has not earlier than been used to maximise the segmental features of female humans. The optimization manner is installation with the objective function (Eq. (9)) and constraints (Eq. (10)):

- Variable size: 30.
- Swarm size: 100.
- Tolerance limit: 0.0001.
- Iterations: 50.
- Mutation coefficient: 0.2^33^.
- Light absorption coefficient: 0.8^33^.

**Fig. 2:**
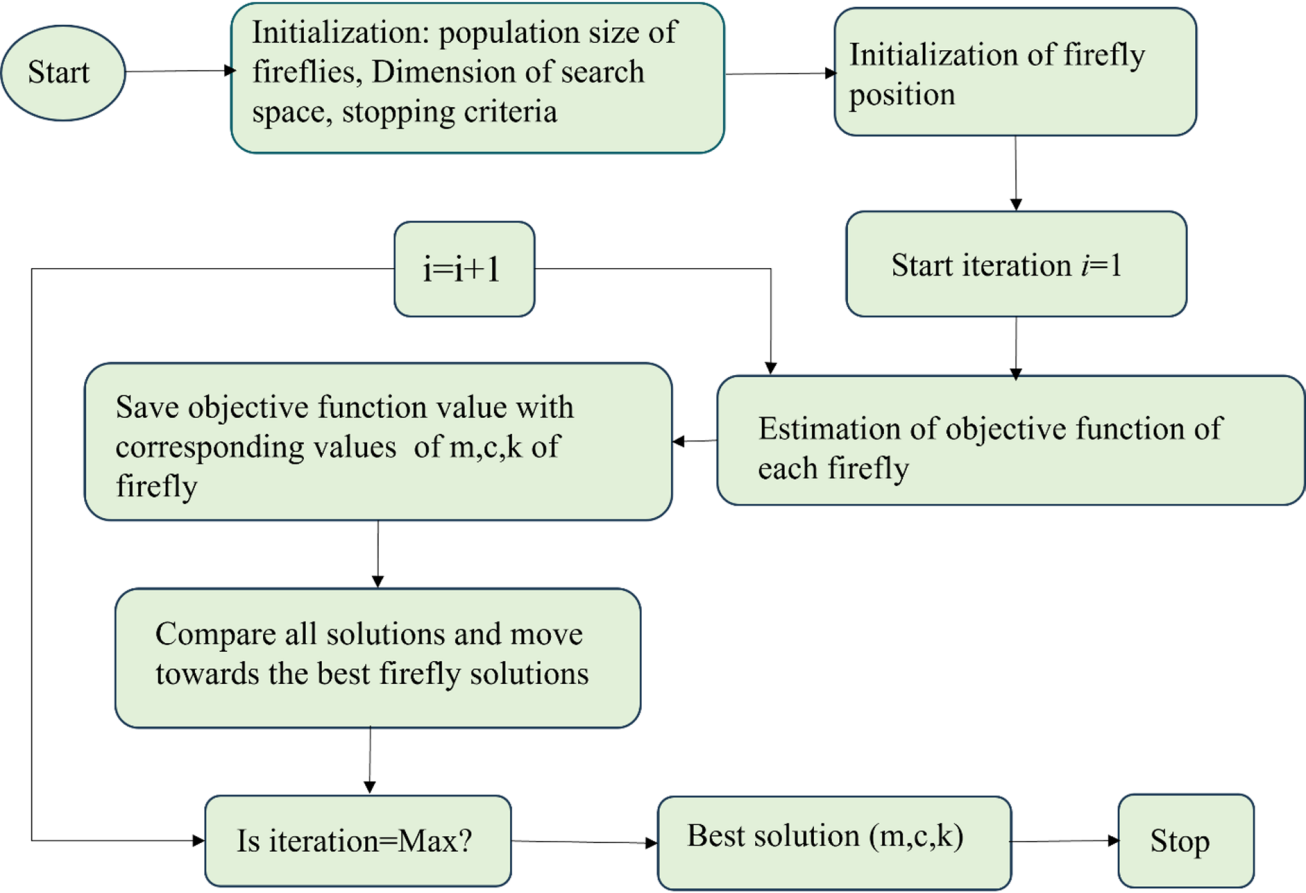
Flow chart for a firefly algorithm to optimize female biomechanical parameters.

**Fig. 3:**
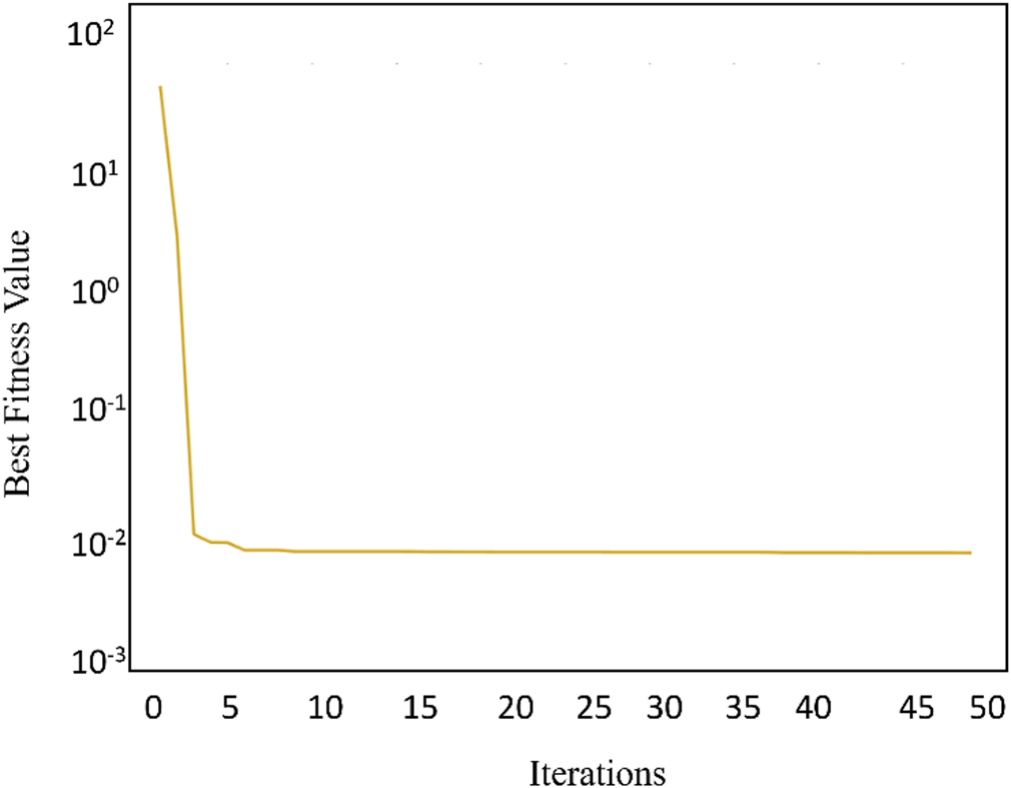
Firefly algorithm convergence plot.

The overall FA optimization process workflow is depicted in Fig. 2, detailing the sequential stages from parameter initialization to solution convergence. As a result of this process, the partially optimized mechanical parameters, such as mass, stiffness and damping values obtained through the algorithm are summarized in Tables 1, 2 and 3, respectively, providing insight into the improved parameter values achieved after iterative refinement. Furthermore, the convergence behaviour of the optimization procedure is shown in Fig. 3, illustrating the progressive reduction in the objective function value as the algorithm approaches an optimal solution.

After optimization, statistical parameters such as goodness of fit (GF), average goodness of fit (AGF), overall goodness of fit (OGF), and variation are computed for every iteration, with partial findings in Table 4. The Sixth iteration achieves the lowest variance (0.00001) and the highest overall goodness of fit (0.968). The corresponding goodness of fit values for DPMI, STHT, and AM are 97.2, 96.8, and 96.5, respectively.

The goodness of fit calculations are given by^19^:

11 $$\varepsilon =1 - \frac{{\sqrt {\Sigma {{({\tau _e} - {\tau _p})}^2}/(N - 2)} }}{{\Sigma {\tau _e}/N}}$$

12 $$\bar {\varepsilon }={\lambda _1}.{\varepsilon _{STHT}}+{\lambda _2}.{\varepsilon _{DPMI}}+{\lambda _3}.{\varepsilon _{AM}}$$

13 $${\bar {\varepsilon }_{MAX}}=Max({\lambda _1}.{\varepsilon _{STHT}}+{\lambda _2}.{\varepsilon _{DPMI}}+{\lambda _3}.{\varepsilon _{AM}})$$

where:

- ${\tau _e}$and ${\tau _p}$are experimental and model data points,
- N has the number of data points, correspond to DPMI, STHT, and AM, respectively,
- λ1 = λ2 = λ3 = 1/3​ (equal weighting).

The weighting function (λ) shows a critical role in optimization by defining the relative importance of biodynamic responses such as driving point mechanical impedance (DPMI), seat-to-head transmissibility (STHT), and apparent mass (AM) in the objective function. Since the objective function minimizes the sum of squared errors across multiple biodynamic responses, the weighting function adjusts how much each response contributes to the final error value. If equal weighting is used (λ₁ = λ₂ = λ₃ = 1/3), all three responses are treated as equally important. However, if a higher weight is assigned to STHT (λ₁ > λ₂, λ₃), the optimization will prioritize minimizing STHT error, potentially at the expense of DPMI and AM accuracy. In the present study, equal significance is allowing to all biodynamic responses to maintain a balanced model.

The weighting function also impacts optimization convergence and accuracy. Assigning higher weights to specific responses can improve model accuracy for those responses but may increase the overall sum of squared errors by neglecting others. Conversely, lowering the weight of certain responses allows the optimization to focus on more sensitive or critical parameters. For example, if human sensitivity to vibration is more strongly linked to STHT, a larger λ₁ might be preferable. Unequal weighting can also lead to faster convergence if the optimization focuses on the most influential parameters first, effectively reducing overall error.

A critical trade-off exists between model generalization and specialization. Uniform weighting (λ₁ = λ₂ = λ₃) creates a balanced model that performs well across all biodynamic responses, whereas non-uniform weighting (λ₁ > λ₂, λ₃) results in a specialized model that excels in one metric but may underperform in others. The choice of weighting function should align with the specific application. In general biomechanical modelling, equal weights are often used, but in application-specific models such as automotive seat design or medical assessments, different weight distributions may be necessary to prioritize certain responses.

Increasing the load of a selected parameter while prioritizing it ensures that the optimization procedure concentrates more on minimizing its mistake than the others. For example, increasing λ₁ (λ₁ > λ₂, λ₃) can enhance STHT accuracy, probably on the rate of DPMI and AM accuracy, if STHT is the maximum crucial factor for human comfort in vehicle seats. However, since STHT, DPMI, and AM are interdependent, overly prioritizing one may degrade the accuracy of the others. A moderate adjustment, such as λ₁ = 0.5, λ₂ = 0.25, and λ₃ = 0.25, would allow reasonable accuracy in DPMI and AM while focusing on STHT.

A well-chosen weighting function is also essential for maintaining convergence and stability in optimization. Properly assigned weights prevent the model from overfitting to less important parameters, whereas poorly assigned weights may cause instability, requiring more iterations and increasing computational time. The ideal weighting function varies by application; for vehicle seat design, prioritizing STHT (λ₁ > λ₂, λ₃) enhances human comfort, while for impact injury analysis, prioritizing AM (λ₃ > λ₁, λ₂) improves force-acceleration predictions. In ergonomic tool design, balancing DPMI and STHT (λ₂ ≈ λ₁ > λ₃) optimizes force distribution and minimizes vibrations. Ultimately, selecting an appropriate weighting function is essential for achieving accurate, stable, and application-specific optimization results. The selection of control parameters for the Firefly Algorithm, such as the light absorption coefficient (γ = 0.8) and the mutation coefficient (α = 0.2), is based on previous studies which indicate that they can effectively ensure a balance between exploration and exploitation in multimodal optimization problems. A higher absorption coefficient (γ) accelerates convergence by limiting the attraction range, preventing excessive random wandering, while a moderate mutation coefficient (α) maintains population diversity, helping to avoid premature convergence. The chosen parameter values are all within the optimal tuning ranges reported in the literature for biomechanical and structural optimization applications (0.6 ≤ γ ≤ 1.0, 0.1 ≤ α ≤ 0.3) [Yang, 2008; Fister et al., 2013; Gandomi et al., 2013]. These settings were further validated through preliminary sensitivity tests, which indicated that γ = 0.8 and α = 0.2 achieve stable convergence and the minimum variance in fitness indicators without overfitting.

**Table 1:** Optimized mass values of female human beings (partial results).

Iteration	Mass (kg)									
	m_1_	m_2_	m_3_	m_4_	m_5_	m_6_	m_7_	m_8_	m_9_	m_10_
1	6.91	16.19	10.80	10.25	2.87	1.44	0.62	2.87	1.44	0.62
2	7.26	17.00	11.34	10.76	3.01	1.51	0.65	3.01	1.51	0.65
3	7.61	17.81	11.88	11.27	3.15	1.58	0.68	3.15	1.58	0.68
4	7.95	18.62	12.42	11.78	3.30	1.65	0.71	3.30	1.65	0.71
5	8.30	19.43	12.96	12.29	3.44	1.73	0.74	3.44	1.73	0.74
6	8.64	20.24	13.50	12.81	3.59	1.80	0.77	3.59	1.80	0.77
7	8.99	21.05	14.03	13.32	3.73	1.87	0.80	3.73	1.87	0.80
8	9.33	21.86	14.57	13.83	3.87	1.94	0.83	3.87	1.94	0.83
9	9.68	22.67	15.11	14.34	4.02	2.01	0.87	4.02	2.01	0.87
10	10.03	23.48	15.65	14.86	4.16	2.09	0.90	4.16	2.09	0.90

**Table 2:** Optimized values of stiffness values of female human beings (Partial Results).

Iteration	Stiffness parameters (Nm^− 1^, ×10^3^)									
	k_1_	k_2_	k_3_	k_4_	k_5_	k_6_	k_7_	k_8_	k_9_	k_10_
1	210.30	160.50	250.00	820.00	260.00	170.00	280.00	260.00	170.00	280.00
2	220.45	165.80	240.12	790.34	250.15	165.00	270.14	250.15	165.00	270.14
3	230.60	171.10	230.24	760.68	240.30	160.00	260.28	240.30	160.00	260.28
4	240.75	176.40	220.36	731.02	230.45	155.00	250.42	230.45	155.00	250.42
5	250.90	181.70	210.48	701.36	220.60	150.00	240.56	220.60	150.00	240.56
6	261.05	187.00	200.60	671.70	210.75	145.00	230.70	210.75	145.00	230.70
7	271.20	192.30	190.72	642.04	200.90	140.00	220.84	200.90	140.00	220.84
8	281.35	197.60	180.84	612.38	191.05	135.00	210.98	191.05	135.00	210.98
9	291.50	202.90	170.96	582.72	181.20	130.00	201.12	181.20	130.00	201.12
10	301.65	208.20	161.08	553.06	171.35	125.00	191.26	171.35	125.00	191.26

**Table 3:** Optimized values of damping values of female human beings (Partial Results).

Iteration	Damping parameters (Nsm^− 1^, ×10^2^)									
	c_1_	c_2_	c_3_	c_4_	c_5_	c_6_	c_7_	c_8_	c_9_	c_10_
1	32.10	23.00	26.50	14.30	22.00	40.00	25.00	22.00	40.00	25.00
2	30.90	22.50	25.40	14.00	21.50	38.00	24.00	21.50	38.00	24.00
3	29.70	22.00	24.30	13.70	21.00	36.00	23.00	21.00	36.00	23.00
4	28.50	21.50	23.20	13.40	20.50	34.00	22.00	20.50	34.00	22.00
5	27.30	21.00	22.10	13.10	20.00	32.00	21.00	20.00	32.00	21.00
6	26.10	20.50	21.00	12.80	19.50	30.00	20.00	19.50	30.00	20.00
7	24.90	20.00	19.90	12.50	19.00	28.00	19.00	19.00	28.00	19.00
8	23.70	19.50	18.80	12.20	18.50	26.00	18.00	18.50	26.00	18.00
9	22.50	19.00	17.70	11.90	18.00	24.00	17.00	18.00	24.00	17.00
10	21.30	18.50	16.60	11.60	17.50	22.00	16.00	17.50	22.00	16.00

**Table 4:** Goodness of fit values (Partial Results).

Iteration number	${\varepsilon _{STHT}}$	${\varepsilon _{DPMI}}$	${\varepsilon _{AM}}$	$\bar {\varepsilon }$	Variance
1	0.874	0.861	0.942	0.892	0.0018
2	0.900	0.910	0.950	0.920	0.0012
3	0.925	0.930	0.960	0.938	0.0009
4	0.950	0.955	0.968	0.958	0.0005
5	0.965	0.965	0.970	0.967	0.0003
6	0.965	0.968	0.981	0.9712	0.0001
7	0.965	0.965	0.970	0.967	0.0003
8	0.950	0.955	0.968	0.958	0.0005
9	0.925	0.930	0.960	0.938	0.0009
10	0.900	0.910	0.950	0.920	0.0012

Table 5 compares the performance of existing male models with a newly proposed female model based on three goodness-of-fit metrics: DPMI, STHT, and AM, along with an overall score. The existing models, including those by Allen, Wan and Schimmels, Bai et al., Darling et al., and Guruguntla and Lal, serve as benchmarks, with Guruguntla and Lal’s model showing the strongest performance among them (97.20 in STHT, 96.80 in DPMI and overall, and 96.5 in AM). However, the proposed female model outperforms all of them, achieving scores of 96.5 (STHT), 96.8 (DPMI), and 98.1 (AM), with the highest overall goodness-of-fit score of 97.12. This indicates that the new model not only matches but exceeds the performance of previous models, particularly excelling in the AM metric. The results suggest that the proposed female model is a significant improvement, offering better accuracy and reliability, which could make it a preferred choice in future applications. The superior performance may stem from refinements in design or methodology, addressing limitations present in earlier male models. Overall, the findings highlight the advancement represented by the proposed female model in this field of study.

**Table 5:** Comparison of existed male and proposed female models.

Model	Goodness of fit			Overall goodness of fit
	STHT	DPMI	AM	
Allen^14^	83.7	55.9	83.2	74.20
Wan and Schimmels^16^	91	80	86.8	85.90
Bai et al.^18^	93.4	91.90	89.8	91.90
Darling et al.^22^	90.84	81.33	87.80	86.66
Guruguntla and Lal^18^	97.20	96.80	96.5	96.80
Proposed Female Model	96.5	96.8	98.1	97.12

**Fig. 4:**
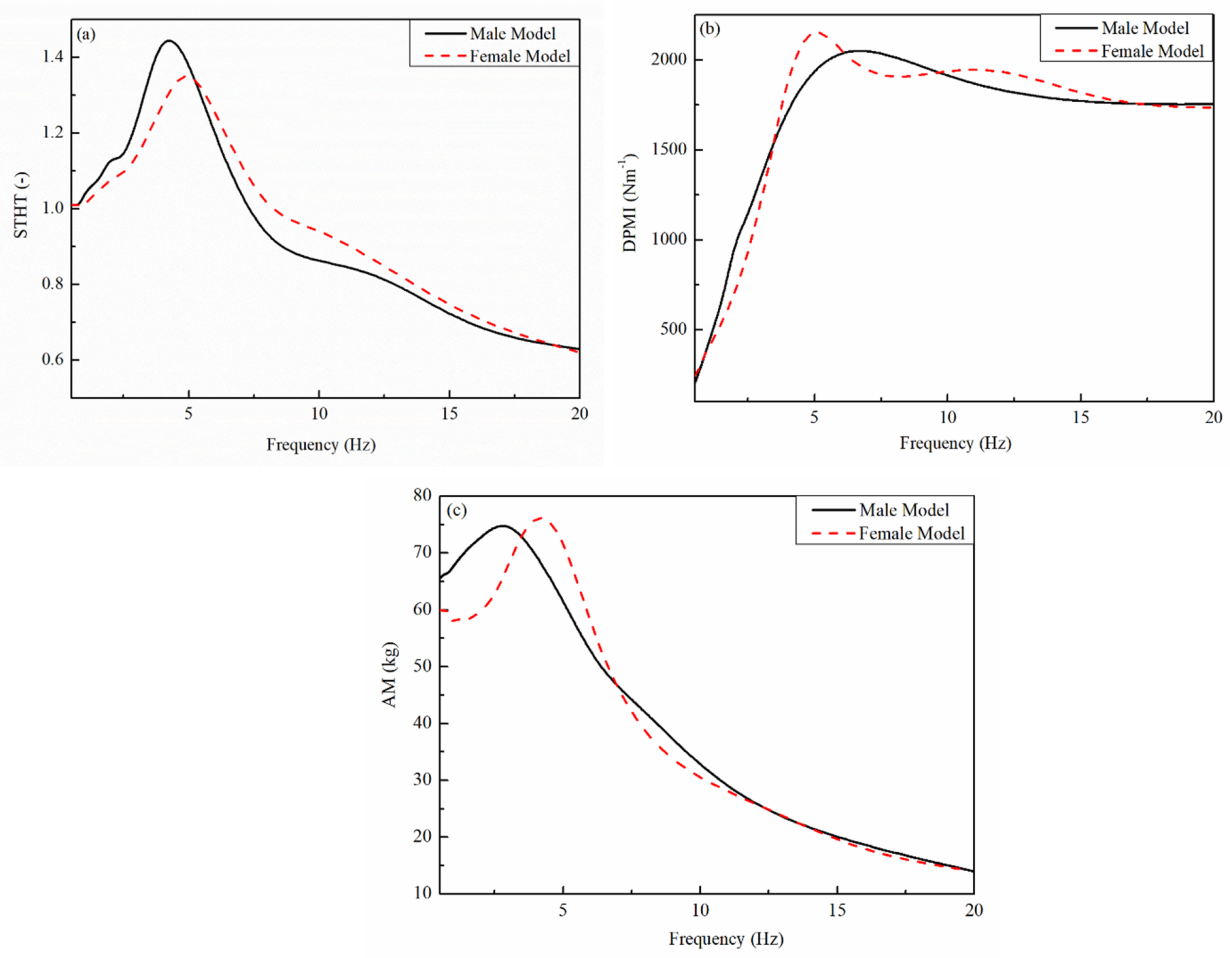
Comparison of male model (Guruguntla and Lal^28^) with female (proposed) biomechanical model.

**Fig. 5:**
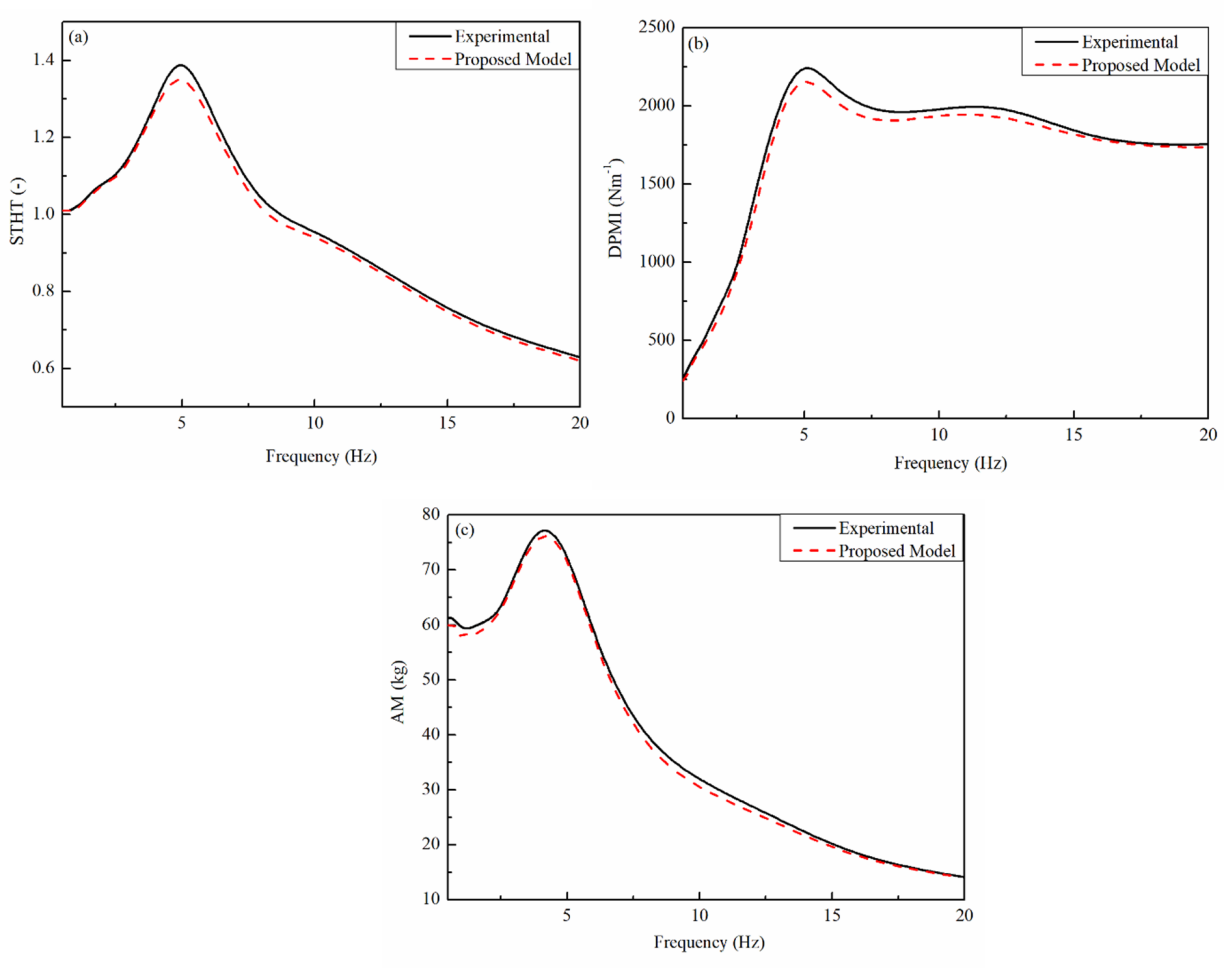
Comparison of experimental data with female (proposed) biomechanical model.

Figures 4 and 5 presents a comparative analysis between experimental data and a proposed model concerning human body response to vibrations at different frequencies. The X-axis in all three subplots represents frequency (Hz), while the Y-axis represents different biomechanical response parameters: Driving Point Mechanical Impedance (DPMI), Seat-to-Head Transmissibility (STHT), and Apparent Mass (AM). Each plot consists of two curves-one representing experimental data (solid black line) and the other representing the proposed model (dashed red line). This image comparison aids in determining how properly the recommended version resembles real experimental findings.

STHT is displayed as a characteristic of frequency in the Fig. 5(a). The transmission of vibrations since the seat to the head is measured through STHT. Both curves on this figure exhibit a comparable pattern, with a remarkable peak at 4–5 Hz suggesting a resonance effect. Due to the human body`s inherent frequency while seated, this peak suggests that the STHT response is maximum sensitive to vibrations on this frequency range. The recommended model quite underestimates vibration transmission at this resonance frequency, as evidenced through the experimental data’s slightly larger peak. Both curves, however, almost suit above 5 Hz, demonstrating that the model correctly forecasts vibration transmissibility at higher frequencies.

DPMI is represented through Fig. 5(b), which measures the body`s resistance to vibratory forces on the seat interface. Like the STHT plot, the DPMI suggests a peak at approximately 4–5 Hz, indicating a body’s frequency-based impedance response. The recommended model understates the mechanical impedance at resonance, even as the experimental data presents a large peak. The curves display a robust affiliation regardless of this small difference, specifically in better frequency stages in which they nearly overlap. Although moderate changes can be required for optimum accuracy, the robust agreement shows that the suggested model correctly depicts the body`s impedance conduct at some stage in vibrational exposure.

AM as a feature of frequency is depicted in the Fig. 5(c), which indicates how the obvious mass of the body modifications in reaction to vibrational input. Since it explains how numerous frame elements reply to outside stimuli at exceptional frequencies, obvious mass is a critical biomechanics quantity. The resonance sample found in the previous subplots is showed through the graph’s distinguished peak, which seems at approximately 4–5 Hz. There are particularly minor variations in the peak area among the experimental and recommended model curves, which agree properly over the majority of the frequency range. Overall, the model gives a close approximation to actual human response data, albeit at better frequencies, it particularly underestimates the experimental values.

All matters considered, the three subplots display that the recommended model correctly reproduces the experimental findings, especially in relation to standard developments and better frequency behaviour. Even with some moderate variations in peak values, specifically in the area of the resonance frequency (4–5 Hz), the model continues to be a manageable depiction of ways the human body reacts to vibration. The model`s ability usefulness in real-world applications inclusive of ergonomic design, vehicle seat optimization, and human frame vibration evaluation is highlighted through the tight correspondence among the experimental information and the model. In order to make certain even better dependability in biomechanical investigations and engineering applications, future model enhancements may want to deal with higher capturing height values and improving its accuracy specially frequency ranges.

**Fig. 6:**
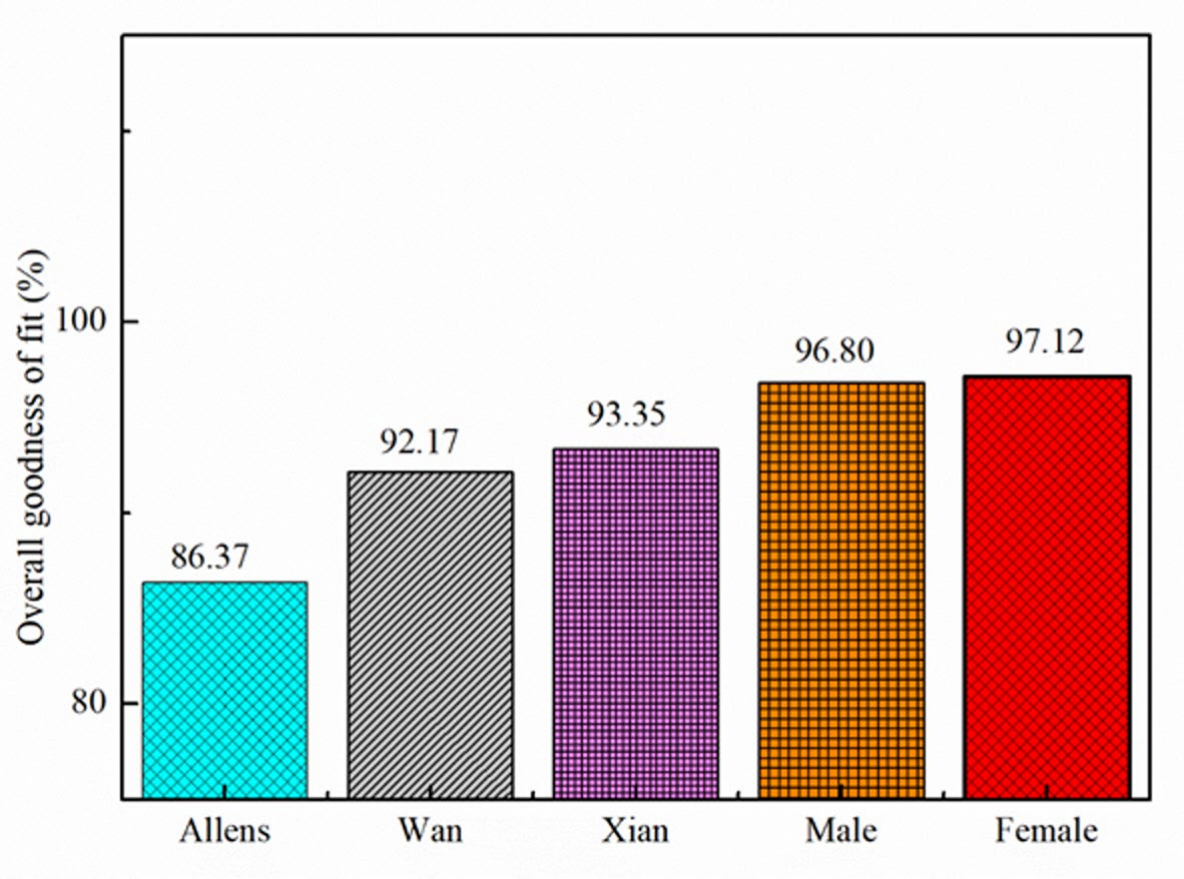
Comparison of male and female biomechanical model GOF.

Figure 6 compares diverse biomechanical models consistent with their normal goodness of fit (%), that is shown at the Y-axis. The Allens, Wan, Xian, Male, and Female (Proposed) Models are the 5 models which can be proven at the X-axis. The overall performance of those models in terms of the way properly they fit the facts is proven through each bar. The findings display that the female model has the best goodness of fit (97.12%), with the male model (Guruguntla and Lal^28^ coming in second at 96.80%. With corresponding goodness-of-fit ratings of 93.35% and 92.17%, the Xian and Wan fashions likewise exhibit robust overall performance. With the lowest fit of 86.37%, the Allens model might not be as right because the others at capturing the underlying biomechanical features.

The efficacy of every model is verified through the specific bar heights; greater values propose higher alignment with the expected results. The small variant among the male and female models increases the opportunity that gender-precise variables may want to make contributions to higher biomechanical forecasts. Traditional models like Wan and Xian, on the opposite hand, do now no longer outperform the gender-based models, however they do perform alternatively well. The poor goodness-of-fit score of the Allens model shows that its assumptions or computational method can be flawed. All matters considered, this figure emphasizes how critical it is to select the proper biomechanical models so that it will assure specific forecasts. According to the data, gender-based models-each male and female-produce higher consequences than traditional models. In domains like ergonomics, clinical diagnostics, and damage prevention, wherein specific biomechanical modelling is critical for efficient evaluation and decision-making, this will have predominant ramifications.

**Fig. 7:**
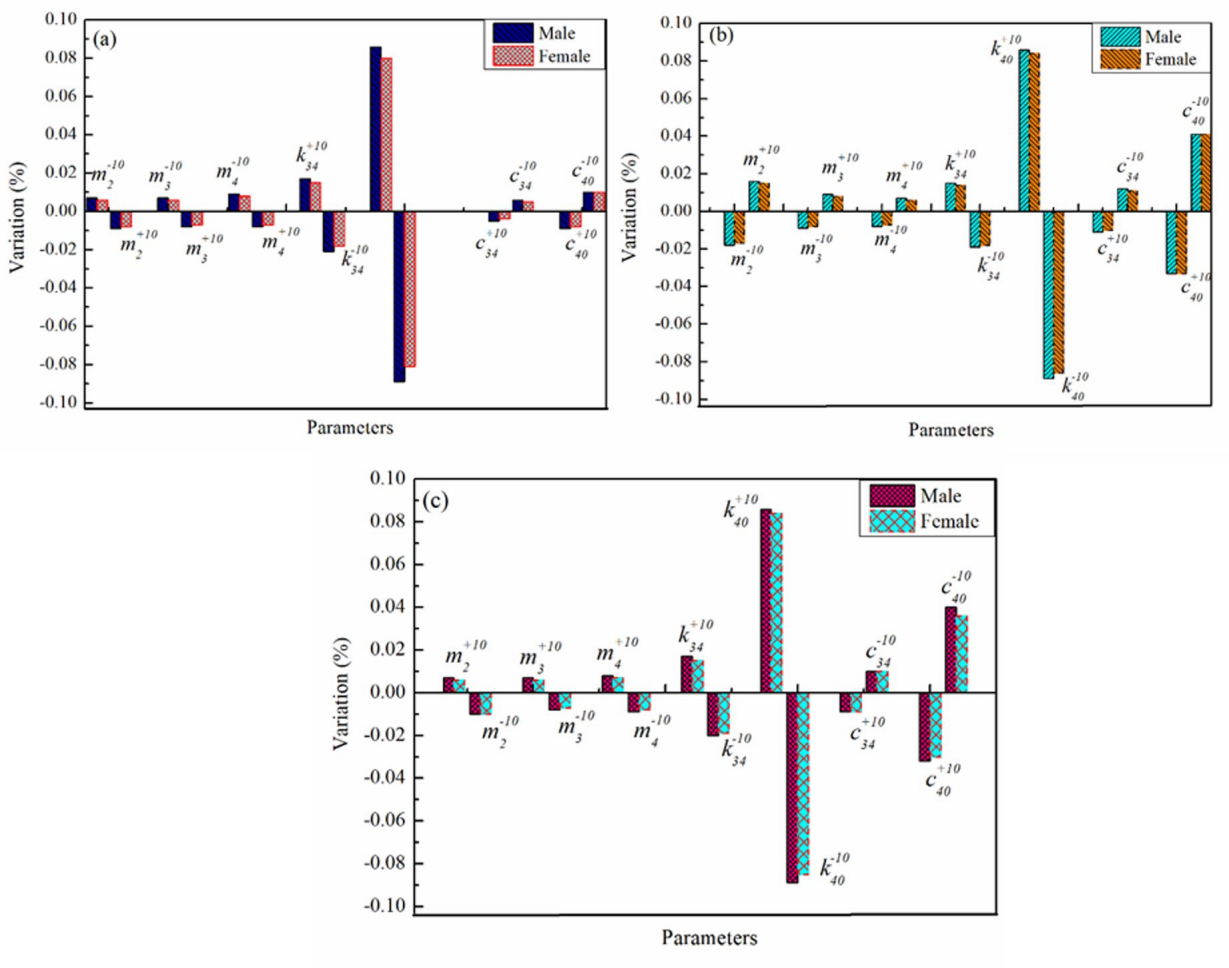
Variation of biodynamic responses (%) For Male (Guruguntla and Lal^28^ and Female (Proposed Model) (**a**) STHT (**b**) DPMI (**c**) AM.

The comparative biodynamic responses are presented in Fig. 7, illustrating differences between male data from Guruguntla and Lal^28^ and female responses from the proposed model. The variation in STHT is shown in Fig. 7(a), while Fig. 7(b) presents the corresponding changes in DPMI. The acceleration magnification results are provided in Fig. 7(c), summarizing the overall response differences between the two groups.

**Fig. 8:**
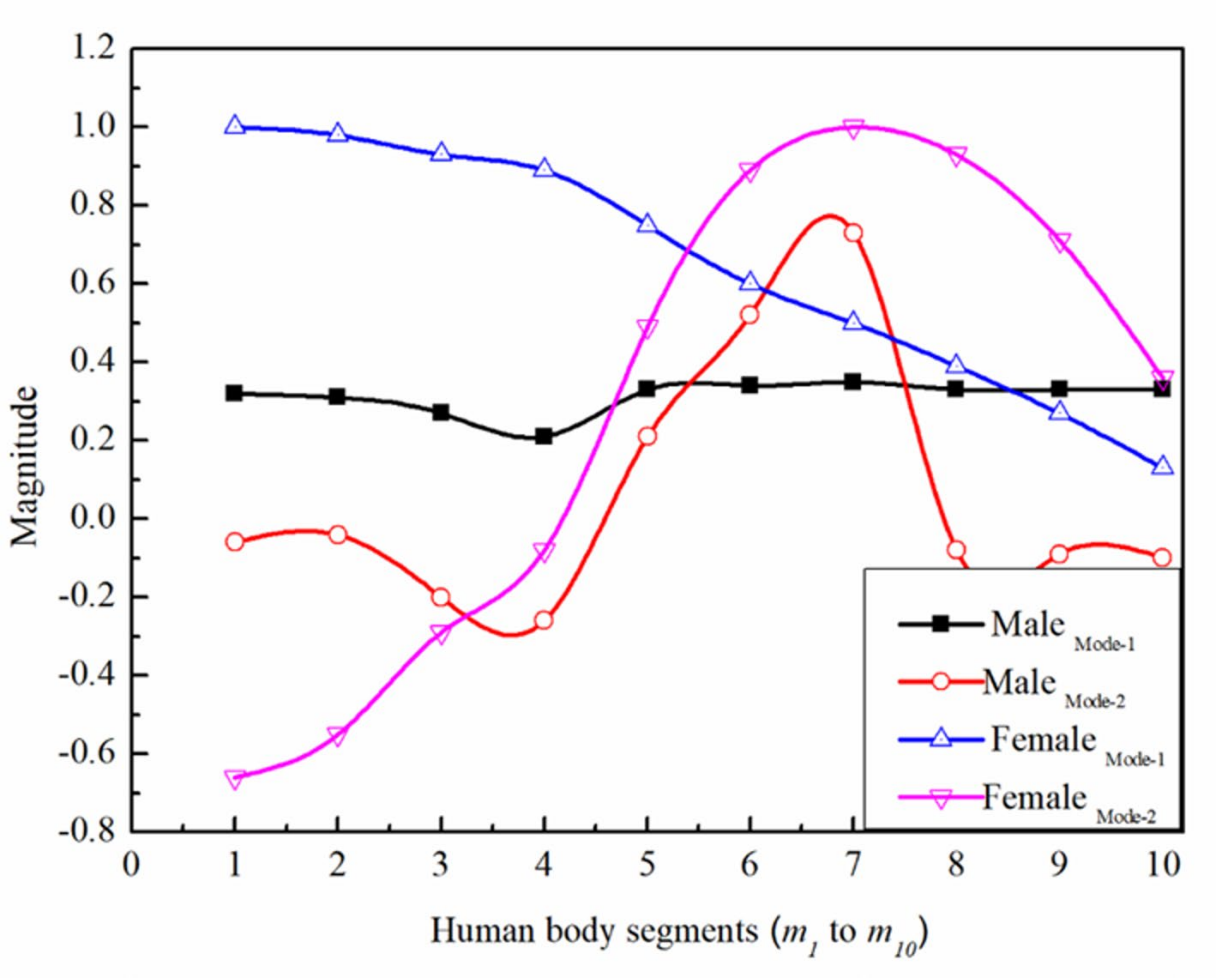
A comparison of first two mode shapes of Male (Guruguntla and Lal^28^) and Female (Proposed Model).

Figure 8 offer an in-depth comparison for natural frequencies and mode shapes of male and female biomechanical models. This visual comparison highlights how the structural and anthropometric differences between the two models influence their vibrational characteristics. The detailed modal properties, including natural frequencies and associated eigenvectors (mode shapes), are summarized in Tables 6 and 7 for ten vibration modes in each model. These values clearly demonstrate noticeable variations in the dynamic responses of the male and female systems, reflecting differences in segment mass distribution, stiffness characteristics, and overall biomechanical configuration.

For the male biomechanical model, the natural frequencies are higher, ranging from 5.48 Hz to 34.43 Hz across the ten modes. The corresponding eigenvectors show consistent amplitude patterns for different nodes in the model, indicating the degree and direction of displacement during each mode. Notably, the third and fourth modes exhibit significantly higher frequencies (over 30 Hz), suggesting stiffer structural characteristics or tighter coupling between elements in the male system.

**Table 6:** Natural frequency and mode shape of the male Biomechanical model of Guruguntla and Lal^28^.

Modes	1	2	3	4	5
Natural frequency (Hz)	5.48	22.23	32.42	33.45	34.43
Eigenvectors	− 0.34	− 0.24	− 0.39	− 0.47	0
	− 0.33	− 0.13	0	0.01	0
	− 0.26	0.43	0.66	− 0.08	0
	− 0.12	0.66	− 0.6	0.06	0
	− 0.33	− 0.17	− 0.03	0.14	− 0.15
	− 0.33	− 0.22	− 0.07	0.34	− 0.40
	− 0.34	− 0.25	− 0.10	0.47	− 0.56
	− 0.33	− 0.17	− 0.03	0.14	0.14
	− 0.33	− 0.22	− 0.07	0.35	0.39
	− 0.34	− 0.25	− 0.10	0.49	0.55

**Table 7:** Natural frequency and mode shape of the female (proposed) Biomechanical model.

Modes	1	2	3	4	5
Natural frequency (Hz)	3.75	11.22	20.75	26.90	33.58
Eigenvectors	1	− 0.66	0.75	0.6	0.31
	0.98	− 0.55	0.31	0.01	− 0.17
	0.93	− 0.29	− 0.48	− 0.78	− 0.48
	0.89	− 0.08	− 0.84	− 0.82	− 0.23
	0.75	0.49	− 1	0.34	1
	0.60	0.89	− 0.3	0.92	− 0.03
	0.50	1	0.27	0.45	− 0.63
	0.39	0.93	0.67	− 0.43	− 0.24
	0.27	0.71	0.75	− 1	0.54
	0.13	0.36	0.36	− 0.79	0.69

In contrast, the female biomechanical model shows lower natural frequencies, from 3.75 Hz to 33.58 Hz, pointing toward a system that may be less stiff or differently configured in mass and damping distribution. Interestingly, the eigenvectors reveal more pronounced variations in modal amplitudes and more complex vibrational behaviour across modes. For example, Mode 3 shows a strong change in displacement direction across nodes, possibly reflecting different dynamic responses due to anatomical or structural distinctions.

Figure 8 and the accompanying plot visualize the first two mode shapes for both male and female models, emphasizing the contrast. The first mode shape (black for male, blue for female) suggests a relatively uniform response for the male system with small oscillations, while the female model shows a gradually decreasing magnitude, indicative of greater deformation or flexibility at one end. The systems are similarly prominent through the second one mode shape (pink for female, red for male); the female reaction has a sharper and more spatially variable mode, while the male reaction has a smoother, symmetrical mode.

Overall, the data display that, even though the female model is much less stiff overall, as indicated through natural frequencies, it responds with more flexibility and complexity in mode shapes. Applications in which unique depiction of human body dynamics is crucial, like biomechanical simulations, clinical diagnostics, and ergonomics, rely upon those variations in modal behaviour.

The developed model and research findings can be applied to ergonomic seat design to optimize comfort and reduce discomfort for drivers and passengers caused by vibration. In addition, the predictive capabilities of this model can be extended to the field of medical diagnosis, particularly for assessing musculoskeletal or spinal health issues related to whole-body vibration exposure. At the same time, the insights gained from this study contribute to the calibration of crash test dummies, thereby improving the accuracy of biomechanical response simulations under various vibration and impact conditions. By outlining these applications, this study not only demonstrates its scientific value but also highlights its potential contributions to safety-, comfort-, and health-oriented engineering innovations.

## Conclusion and future scope

The study efficiently created and subtle a 10-dof female-unique biomechanical model for analysing biodynamic reactions in the presence of vertical vibration. Utilizing the Firefly Algorithm (FA), the model turned into subtle to exactly forecast critical biodynamic parameters, which include Driving Point Mechanical Impedance (DPMI), Apparent Mass (AM), and Seat-to-Head Transmissibility (STHT), and it outperformed contemporary male-orientated models. Notably, the proposed female model achieved the highest overall goodness of fit (97.12%), outperforming well-established male models such as that by Guruguntla and Lal^28^ (96.80%) and others.

The study highlighted the significant role of gender-specific anatomical differences-including lower body mass, higher soft tissue damping, and distinct mass distribution-in shaping vibration responses, thereby underscoring the necessity for gender-tailored modelling approaches. Modal analysis further revealed that the female model exhibited lower natural frequencies (3.75–33.58 Hz) and more intricate mode shapes compared to male counterparts (5.48–34.43 Hz), reflecting fundamental differences in stiffness and damping properties.

The model’s great accuracy was validated experimentally against real-world data, especially in the crucial 4–5 Hz frequency region where human sensitivity to vibration is most noticeable. These results highlight how crucial it is to take gender-specific biomechanical traits into account when designing ergonomic systems, car safety features, and vibration reduction techniques.

Several opportunities exist for expanding the scope of this research in the future. The model could be enhanced to account for demographic variability, including factors such as age, body mass index (BMI), and pregnancy, thereby extending its applicability to a wider population. Multi-axis vibration analysis, incorporating lateral, fore-aft, and rotational motions, should also be considered to more accurately reproduce real-world environments such as off-road driving or aircraft vibration conditions. Additionally, hybrid optimization techniques coupling FA with machine learning algorithms may be employed to further refine model parameters while reducing computational overhead.

The model also has significant potential for integration into intelligent adaptive systems, such as active seat suspension platforms with real-time damping control, improving safety and comfort across users. Moreover, it can support the study of vibration-induced health problems, including spinal disorders, and contribute to the development of preventive strategies for high-risk professions such as pilots and truck drivers. Beyond clinical and occupational applications, the model may aid in designing female-specific crash test dummies and enhancing safety systems such as airbags and seatbelts. Finally, large-scale experimental studies involving diverse participant groups will be essential to validate the model’s robustness and expand its relevance to various operational environments and user conditions.

## Data Availability

All relevant data, simulation files, and figures supporting the findings of this study are included within the manuscript. No additional external datasets are required to reproduce the results.
